# Focal therapy for prostate cancer in Ireland: addressing the national objective and subjective needs

**DOI:** 10.1007/s11845-025-04023-1

**Published:** 2025-07-26

**Authors:** Robert A. Keenan, Kevin Keane, Jody Khan, Muhammad Z. Ahmed, John F. Sullivan, Rustom P. Manecksha, Louise C. McLoughlin, Peter E. Lonergan, Mohammed S. Inder

**Affiliations:** Department of Urology, St. James’s Hospital, James’s Street, Dublin 8, Ireland

**Keywords:** Focal therapy, Healthcare management, Healthcare planning, Prostate cancer

## Abstract

**Introduction:**

Focal therapy has been approved by both NICE and EAU for localised prostate cancer treatment as part of a prospective registry; however, it is not yet available in Ireland. This study aims to assess the proportion of patients with prostate cancer who are suitable for this treatment and to assess attitudes amongst specialists in Ireland.

**Methods:**

All patients with a new prostate cancer diagnosis in a tertiary-referral unit within a 12-month period had their radiological and clinicopathological parameters examined by an expert in the delivery of focal therapy to assess their suitability for treatment. Secondly, a questionnaire addressing current knowledge and attitudes was sent to all consultant urologists and trainees nationally.

**Results:**

Two hundred seven patients were seen in a prostate cancer diagnosis clinic in 2023. Following exclusions, of the 191 patients with clinically localised disease, 50% (*n* = 96: 8% GG1; 71% GG2, 16% GG3, 3% GG4) are technically suitable and 27% (*n* = 52: 6% GG1; 69% GG2, 19% GG3, 6% GG4) are ideal candidates for focal therapy. 23/28 trainees and 25/72 consultants responded to the survey (48%). Ninety-four percent routinely deal with prostate cancer diagnoses; however, almost 90% do not inform patients focal therapy is a treatment option. Forty-four percent of trainees and half of consultant prostatectomists are interested in learning focal treatment.

**Conclusion:**

Although there is a lack of exposure amongst Irish urologists, there is considerable interest in introducing focal therapy nationally. Up to half of patients with new prostate cancer diagnoses may be suitable for treatment highlighting the potential for developing this service in Ireland.

## Introduction

Focal therapy has been approved by both NICE and EAU for localised prostate cancer treatment as part of a prospective registry; however, it is not yet available as a treatment option for patients in Ireland. In the low to intermediate risk groups, focal therapy has demonstrated equivalent oncological outcomes up to 8 years [1]. Functional outcomes, in terms of both continence and erectile function as well as recovery from treatment, are improved in these patients making focal therapy an attractive option for men who want oncological control but seek to avoid the toxicity of conventional treatment options [1, 2]. This study aims to make a case for the introduction of focal therapy in Ireland by assessing the proportion of patients with prostate cancer in a contemporary cohort who are suitable for this treatment. Furthermore, we aim to address the attitudes and perceptions of Irish consultant and trainee urologists towards their knowledge of focal therapy and barriers to implementation.

## Methods

To assess the unmet need of focal therapy for patients with prostate cancer, this study had two main processes. All patients with a new prostate cancer diagnosis in the author’s institution are seen in a dedicated clinic to discuss results and treatment options following multidisciplinary discussion of clinical parameters, imaging review and histopathological review and are kept in a prospectively maintained database. All patients reviewed in a 12-month period (1st January 2023 to 31 st December 2023) had their anonymised radiological and clinicopathological parameters examined. Parameters assessed included age, PSA, clinical tumour stage, PIRADS score, tumour location and ISUP grade. Risk stratification was performed using the Cambridge Prognostic Group (CPG) [3]. Review of each patient’s multiparametric MRI in the context of their clinical parameters was performed by the senior author, an expert in the delivery of focal therapy, to assess their suitability both from a technical perspective and in terms of optimum treatment in the context of their disease.

Outcome was determined as (1) ideal candidate, (2) suitable candidate or (3) unsuitable candidate, determined according to criteria set by international expert Delphi consensus and trial data with any discrepancies or individual patient nuances determined by the senior author [4, 5]. Ideal candidates comprised of intermediate risk disease (CPG 2 or 3) with a focal or discrete lesion on MRI and PSA < 20. Patients with CPG 1 are considered prime candidates for active surveillance [6] and although EAU guidelines do not recommend focal treatment for patients with high risk disease (CPG 4/5), patients with clinical T3a disease and ISUP 3 or less are surgically feasible for focal treatment; therefore, both categories are considered technically suitable though not ideal. Patients with nodal or metastatic disease, clinical T3b/T4, ISUP 5, bilateral disease ISUP 2 ≤ or CPG5 are unsuitable.

The second component of the study involved sending a 10-item questionnaire addressing current knowledge and attitudes to all consultant urologists and trainees nationally. The questionnaire addressed objective knowledge regarding the current status of focal therapy as well as indications/complications and subjective assessment of their attitudes towards focal therapy including interest in training and barriers to its implementation.

## Results

Two hundred seven patients were seen in a prostate cancer diagnosis clinic in 2023. In terms of baseline patient characteristics, median age was 66 years (IQR 9 years) and PSA was 6.96 ng/mL (IQR 6.05). Only one patient (0.5%) did not have an MRI due to claustrophobia. The majority of patients (*n* = 157, 75.8%) had clinically localised (cT1/T2) prostate cancer on MRI. One patient (0.5%) had radiological pelvic nodal disease, cN1. Baseline characteristics are shown in Table 1.

**Table 1:** Baseline characteristics of patient cohort

Patient factor	Total – 207 (%)		
**Age**	66 (IQR 9) years	**MRI lesion**	*n* (%)
**PSA**	6.96 (IQR 6.05) ng/mL	PIRADS 2	15 (7.3%)
**MRI stage**	*n* (%)	PIRADS 3	25 (12.1%)
T1/T2	157 (75.9%)	PIRADS 4	69 (33.5%)
T3a	34 (16.4%)	PIRADS 5	67 (32.5%)
T3b	11 (5.3%)	Multifocal PIRADS 3 ≤	26 (12.6%)
T4	3 (1.4%)	Non-diagnostic	1 (0.5%)
N +/M1	1 (0.5%)	Not specified	3 (1.5%)
Not done	1 (0.5%)		
**ISUP disease (highest)**	*n* (%)	**CPG risk group**	*n* (%)
GG1	28 (13.0%)	CPG 1	23 (11.1%)
GG2	107 (51.7%)	CPG 2	72 (34.5%)
GG3	36 (17.4%)	CPG 3	42 (20.3%)
GG4	14 (6.8%)	CPG 4	34 (16.4%)
GG5	22 (10.6%)	CPG 5	36 (17.4%)
Not graded	1 (0.5%)		
Bilateral GG2 ≤	64 (30.9%)		
Not graded	1 (0.5%)		

With regard to treatment decisions, all patients with CPG one disease were managed with active surveillance from diagnosis. Active surveillance comprised 20% (*n* = 14) of patients with CPG two disease whilst approximately two thirds of patients opted for radical prostatectomy or radical radiotherapy. Radiation therapy was the dominant treatment option for patients with higher grade disease with 40% (*n* = 17), 63% (*n* = 22) and 78% (*n* = 28) for CPG groups three, four and five respectively. Of these patients, 16 patients with high-risk disease were also suitable for the addition of abiraterone.

Robotic prostatectomy was an increasingly less favourable option for patients with increasing CPG risk group with 33% (*n* = 14), 21% (*n* = 7) and 6% (*n* = 2) for groups three, four and five, respectively. Three patients in total (1.4%) were referred abroad for focal treatment. Treatment options for each CPG group are shown in Table 2.

**Table 2:** Treatment options for each CPG group

Treatment option	CPG 1 % (*n* of CPG 1)	CPG 2 % (*n* of CPG 2)	CPG 3 % (*n* of CPG 3)	CPG 4 % (*n* of CPG 4)	CPG 5 % (*n* of CPG 5)
Active surveillance	100 (23)	19.4 (14)	9.52 (4)	2.94 (1)	Nil
Robotic prostatectomy	Nil	31.9 (23)	33.33 (14)	20.59 (7)	5.56 (2)
Focal therapy	Nil	2.78 (2)	2.38 (1)	Nil	Nil
Brachytherapy	Nil	4.17 (3)	7.14 (3)	2.94 (1)	Nil
Radical radiotherapy +/− ADT	Nil	34.72 (25)	40.48 (17)	55.88 (19)	41.67 (15)
Radical radiotherapy +/− ADT + Abiraterone	Nil	Nil	Nil	8.82 (3)	36.11 (13)
ADT monotherapy	Nil	1.39 (1)	Nil	Nil	Nil
ADT + abiraterone	Nil	Nil	Nil	2.94 (1)	5.56 (2)
Chemotherapy	Nil	Nil	Nil	Nil	2.78 (1)
Watchful waiting	Nil	2.78 (2)	2.38 (1)	2.94 (1)	5.56 (2)
Undecided or deferred	Nil	1.39 (1)	2.38 (1)	Nil	2.78 (1)
Declined or DNA	Nil	Nil	2.38 (1)	2.94 (1)	Nil

*ADT* androgen deprivation therapy, *CPG* Cambridge Prognostic Group, *DNA* did not attend

Following exclusions, of the 191 patients with clinically localised disease, 50% (*n* = 96: 8% GG1; 71% GG2, 16% GG3, 3% GG4) are technically suitable and 27% (*n* = 52: 6% GG1; 69% GG2, 19% GG3, 6% GG4) are ideal candidates for focal therapy.

**Fig. 1:**
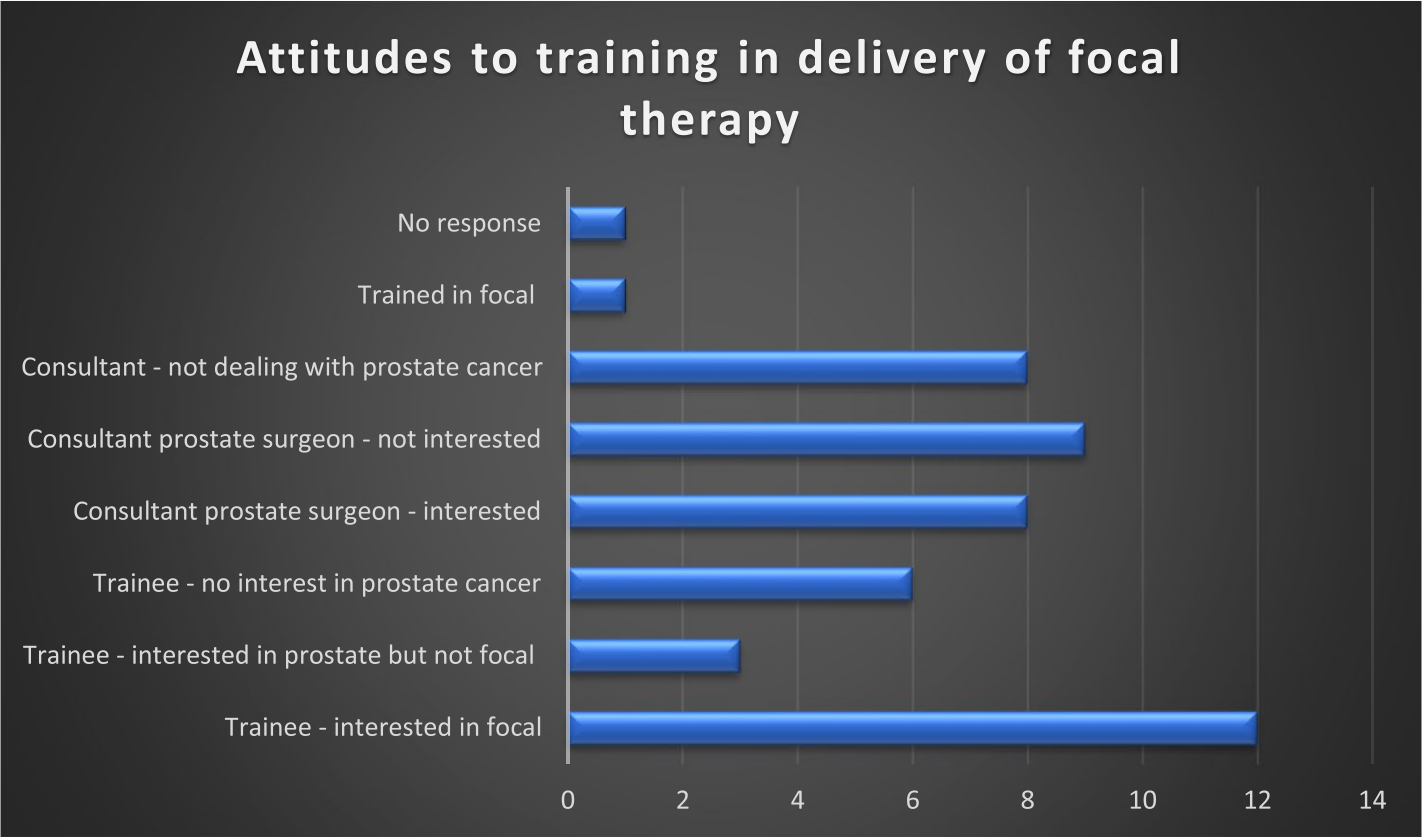
Proportion of respondents interested in training in focal therapy for localised prostate cancer

For the second part of the study, following circulation of a questionnaire, 23/28 (82% response) of all national urology trainees and 25/72 (35% response) of all consultant urologists responded to the survey (48% combined response). Of the 48 respondents, 15 (31%) were consultants who perform radical prostatectomy, 11 (23%) were consultants not performing prostatectomy, two (4%) were fellows, 12 (25%) were urology trainees in ST6 to ST8 training years and eight (17%) were trainees in ST3 to ST5 training years. One of the consultants performing prostatectomy is also fellowship trained in the delivery of focal therapy (Fig. 1).

With regard to familiarity with focal therapy and access to patients with prostate cancer, 39 (81%) have had no clinical exposure to focal therapy as a trainee, fellow or consultant. Forty-five (94%) of respondents routinely deal with prostate cancer diagnoses and counsel patients on treatment options. Twenty-eight (53%) of respondents were aware that focal therapy is now approved by both EAU and NICE as therapeutic options for clinically localised prostate cancer. Twenty-six (54%) are familiar with the differences between the various types of focal therapy, one (2%) did not know the difference and 21 (44%) were vaguely aware but not enough to know the indications or rationale for one modality over another. Eighteen (38%) never discuss focal therapy as a treatment option, 24 (50%) only discuss it as a treatment option if the patient asks about it, three (6%) discuss it sometimes and one (2%) always discusses it as a treatment option.

**Fig. 2:**
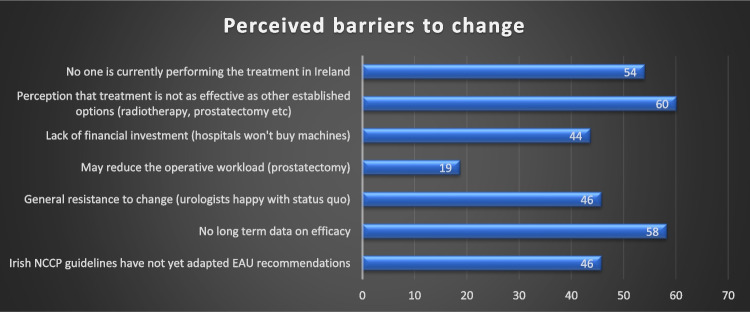
Perceived barriers to the introduction of focal therapy (x-axis—% of respondents)

Regarding implementation in Ireland, 12 (25%) of trainees and eight (29%) of the 28 consultant/fellow responders would consider learning how to deliver focal therapy. A summary of the attitudes towards training is shown in Fig. 2. Responders were allowed to select more than one response. Twenty-two (47%) of urologists who responded would like to see the Irish guidelines and position on focal therapy updated to facilitate the addition of focal therapy as a treatment option for patients with prostate cancer whilst 19 (40%) remain unsure. Only three (6%) did not want this to change.

## Discussion

These results give us an insight into the baseline detailed disease characteristics of patients with prostate cancer attending a regional cancer centre. Based on the focal therapy suitability recommendations as discussed in the methodology, up to 50% of patients with a new prostate cancer diagnosis are suitable for focal treatment whilst 27% are ideal candidates. These results are in keeping with other studies which have assessed potential suitability such as Nassiri et al. who found 38.5% of patients were eligible for focal therapy based on NCCN intermediate risk disease only with no high-risk features and up to ISUP grade 3 disease (+/− ISUP 1 disease, < 4 mm, outside the region of interest) [7]. Other studies such as von Hardenberg et al. have found focal therapy suitability much lower, ranging from 12 to 16% depending on the modality, although have arguably more conservative criteria such as maximum ISUP grade 2 disease and PSA up to 10 [7]. Considering there are approximately 3000 new prostate cancer diagnoses annually in Ireland, when extrapolated, our results imply that over 800 patients are potentially ideally suited to focal therapy as their main form of cancer treatment [8]. Focal therapy is of course competing with radical radiotherapy, brachytherapy and radical prostatectomy for its market share of active treatment options, but the proportion of patients who will ultimately opt for it as their treatment remains unseen.

The exact treatment space for focal therapy is yet to be determined. Some authors suggest it may act as a bridge between rigorous active surveillance protocols and radical treatments such as RP or RRTx [9]. Others have argued that the main objective for low-risk patients is to avoid overtreatment and, although FT is associated with reduced urinary and sexual function consequences, it is not without side effects; therefore, it may still represent overtreatment in this patient cohort [10]. On the other hand, in a review of real-world data best practice statement by Deivasigamani et al., up to 22% of patients being treated with focal therapy were in D’Amico high-risk category [5]. Ultimately, global consensus on the management of prostate cancer with focal therapy has yet to be agreed upon and, although EAU does not currently recommend focal therapy in high-risk clinically localised cases, with increasing volume and long-term data, we may see the criteria and applications for focal therapy expand further with time [6, 11].

When considering introducing new treatment options, when assessed in the NHS, focal therapy is overall cheaper for a health service and also associated with higher QALYs (quality adjusted life years) than radical prostatectomy; therefore, it presents itself as an attractive option for prostate cancer treatment in allowing more patients to be treated for the same budgetary allocation [12]. Optimal success in delivering focal therapy as well as meaningful participation in clinical trials for a population the size of Ireland likely means that focal therapy should ideally be delivered in regional or national centres [7].

From our survey, it is clear there is a significant void in both the knowledge of focal therapy amongst Irish urologists despite the vast majority routinely dealing with prostate cancer diagnoses, as well as a large gap for focal therapy in the prostate cancer treatment paradigm for Irish patients. Although there are many perceived barriers to implementation of focal therapy in Ireland as shown in Fig. 2, it is encouraging to learn that a quarter of trainees and consultant prostatectomists would consider training in focal therapy to be in the position to offer it to Irish patients and only a small percentage of respondents do not want the current Irish guidelines to change. The fact that it is not yet offered in Ireland and is deemed a barrier to implementation by over 50% of respondents is in keeping with findings from a major European survey who found that focal therapy availability influences urologist opinion [13].

Ireland is however not dissimilar to other European counterparts such as Germany where published results have shown that 48% of respondents would not recommend focal therapy due to lack of robust evidence and only one quarter believe focal therapy will occupy the standard treatment space in the future [14]. Attitudes from European and North American urological bodies towards focal therapy have been somewhat mixed however. A survey of American Urology Association (AUA) members showed an increase from 24 to 43% in members utilising focal therapy as a treatment option in the time period between 2017 and 2023 therefore it has certainly increased in popularity [15, 16]. Focal therapy is more likely to be utilised in centres with high-volume new diagnoses (10 per month), fellowship training in uro-oncology and in focal therapy, surgeon experience of > 15 years and “belief” in focal therapy as a treatment option [14–16]. On the other hand, similar barriers exist such as lack of belief in the modality, lack of experience and high cost whilst, interestingly, attendance at international conferences and high volume of high-risk prostate cancer disease are associated with a negative opinion of focal therapy [13, 15, 16].

This study creates a case for the introduction of focal therapy in Ireland by highlighting the real-world application of the treatment outside of trial data in a contemporary cohort and is also the first study to assess the attitudes of Irish urologists and trainees towards the introduction of a new technology in the prostate cancer treatment domain. Going forward, as focal therapy is already established in many centres worldwide including in North America, Europe, the UK and Asia, it will likely find its place in Ireland in the coming years; therefore, this paper provides a baseline from which to compare to. Ireland is in a relatively unique position to be able to introduce focal therapy to a population outside of a trial setting and is an exciting space to watch in terms of future research and establishing long-term outcome data.

Limitations include the fact that the data regarding whether patients are “ideal” or “suitable” candidates is purely theoretical and likely represents a best-case scenario as there are many other factors to consider such as patient preference, treatment availability and treatment cost. At the time of drafting this manuscript, there is only one urologist in Ireland currently trained in the delivery of focal therapy however no hospital is currently supplying the equipment or necessary facilities. This does not create a welcoming environment for current trainees to commit to fellowship training in the technique despite there being considerable interest; therefore, implementation may be slow.

## Conclusion

Although there is a lack of exposure and intricate knowledge of focal therapy amongst Irish urologists, there is considerable interest and a sense of optimism in introducing it as a treatment option for suitable patients with localised prostate cancer. There are many perceived barriers to its implementation including lack of long-term follow-up and that it may not be as effective as other established modalities; however, these are similar to other health systems worldwide that have successfully introduced it and seen growth in recent years. Up to half of patients with new prostate cancer diagnoses may be suitable for treatment, and over one quarter are ideal candidates highlighting the potential treatment space to develop. This poses an exciting opportunity to contribute both to future research in the field and improved functional outcomes in prostate cancer treatment for Irish patients.
